# Population distribution by ethnicities and the disparities in health risk and coping in the United States during the pandemic: the spatial and time dynamics

**DOI:** 10.1186/s13690-022-00858-7

**Published:** 2022-03-25

**Authors:** Jiannan Li, Xinmeng Wang, Bocong Yuan

**Affiliations:** 1 Institute of Advanced Studies in Humanities and Social Sciences, Beijing Normal University, Zhuhai, China; 2 School of Tourism Management, Sun Yat-Sen University, West Xingang Rd. 135, Guangzhou, 510275 China

**Keywords:** Ethnic minorities, Health risk, Vaccination coverage, Population distribution, Pandemic

## Abstract

**Background:**

As a multi-ethnic country, the US is increasingly concerned about ethnic minorities facing disproportionate health risks of the coronavirus disease 2019 (COVID-19) pandemic. This study attempted to provide a macro picture of the associations between population distribution by ethnicity and the vulnerability to COVID-19 in terms of infection risk and vaccination coverage in the US.

**Methods:**

This study used multi-source data from New York Times, County Health Rankings & Roadmap Program (2020), and the Center for Disease Control and Prevention. Multiple linear regressions were performed at equidistant time points (May 2020-Jan 2021, with one-month interval between each time point) to reveal the association between population distribution by ethnicities and the infection risk and the dynamics over time. Besides, multiple linear regressions were also conducted at equidistant time points (Jan 2021-Aug 2021) to reveal whether health disparities between ethnicities would hold true for the COVID-19 vaccination coverage (in total population, and among those > 12, > 18, and > 65 years of age).

**Results:**

Both the COVID-19 confirmed cases (population standardized) and the vaccination coverage (in total population, and among those > 12, > 18, and > 65 years of age) were significantly associated with the population distribution by ethnicity (*e.g., population percentage of ethnic minorities*). Above associations were statistically significant for non-Hispanic blacks and Hispanics, but not for Asian Americans.

**Conclusions:**

A proportion of socioeconomically-disadvantageous population could be a key intuitive reflection of the risk level of this public health crisis. The policy focusing on the vulnerable population is important in this pandemic.

## Background

The burdens of COVID-19 pandemic are not economically and socially equal across different populations. Socioeconomically disadvantaged populations are reported to be at greater risk during the pandemic, with higher rates of infection recorded in impoverished communities, ethnic minorities, the homeless, and people with pre-existing stigmatized infectious disease (e.g., HIV) [1–3]. Prior evidence shows that populations with socioeconomic disadvantages may be more vulnerable to the COVID-19 pandemic and bear a higher risk of morbidity and mortality. For example, it is difficult for poor people to comply with strict sanitation requirements and adopt hygiene tips to protect themselves from the virus, since low-income families are more likely to have less access to clean drinking water and a sufficient supply of sanitizers during the pandemic [4]. Ethnic minority migrants are more likely to live in more crowded, multigenerational housing and thus are less able to keep social distancing [4]. Fewer than a fifth of Black American workers and roughly a sixth of Hispanic workers have the opportunity of telework, compared with nearly 30 percent of non-Latino whites [5]. Black Americans are therefore more likely to work in basic service jobs that require frequent human contact (such as food, delivery, transportation) [5].

These conditions placed ethnic minorities with socioeconomical disadvantages at higher risk of COVID-19 infection, morbidity, and mortality. Long-standing historical discrimination, inadequate access to quality health care, and the economic constraints force these populations to make decisions based on economic reasons rather than giving priority to overall health [6]. Besides, socioeconomic inequities are also associated with population inequities in terms of pre-existing diseases (e.g., diabetes, heart disease, respiratory diseases), which may lead to higher mortality rates among people with pre-existing conditions after contracting COVID-19 [7, 8]. More importantly, inadequate health awareness about COVID-19 also plays a vital role in increasing the risk in these poor communities [9]. Previous study shows a disproportionately high rate of positive tests among blacks and low-income people without health insurance [10]. The disparity in health risks between ethnicities may not be limited to infection. A recent investigation shows that ethnic minorities who have ever been faced with racial discrimination are more likely to be hesitant about vaccination [11]. Long-standing medical mistrust based on racial/ethnic groups may also explain the lower rates of vaccination intake among ethnic minorities [12]. It is reported that non-Hispanic blacks are 11% less likely to be vaccinated than non-Hispanic whites, while Asian Americans are 50% more likely to be vaccinated [13].

Although some efforts have been made by prior studies on this issue, there are still some research gaps. First, most previous studies have focused on individual-level explanations for the risk of COVID-19 infection in specific populations, with using hospitalization data. This study aims to depict, at a macro-level, the associations between population distribution by ethnicity and vulnerability to this pandemic in specific regions. Second, compared with prior studies that show the relationships between population distribution of ethnicities and infection risk at a given timepoint, this study provides the dynamics over time by examining their associations at multiple equidistant timepoints. The examination at a given timepoint lacks sufficient reliability, since the frequency mismatch between population distribution by ethnicity (which is usually stable over several years) and infection/vaccination (which is often volatile and varies from day to day). Third, prior studies have not addressed whether the disproportionate risk of COVID-19 infection faced by ethnic minorities would extend to vaccination coverage. This study fills this research gap by providing an empirical examination.

## Ethnic disparities in infection risk and health care utilization in the pandemic: a review

There are growing concerns that racial/ethnic minorities are at the disproportionate risk of mortality and morbidity of the pandemic. A national cohort study of the veterans in the US finds that the additional risks and burdens experienced by racial minorities could not be entirely explained by healthcare conditions, residence place, or the hospitalized facilities where they access care [14]. Similar results are observed in another study which uses ambulatory care data from Bronx, New York, and reports that blacks have a 1.6 times higher risk of mortality than whites [15]. Massachusetts General Hospital records a rise in Hispanic patients from 9% before the outbreak of pandemic to 35–40% after [16]. A report from Boston Medical Center shows that about 44.7% outpatients with novel coronavirus disease symptoms are non-Hispanic blacks and 14.3% are non-Hispanic whites [14]. Non-Hispanic blacks and non-Hispanic whites account for 42% and 13.4% of severe cases in ICU with mechanical ventilation [17].

The elevated risk for colored people can also be related to the differences in pre-existing comorbidities and testing rates [18]. Ethnic minorities have higher odds of obesity, diabetes, and cardiovascular diseases, which could easily induce comorbidities of novel coronavirus disease, leading to a higher mortality risk [19]. There are also significant racial differences in the awareness, knowledge, and response to novel coronavirus disease, with non-Hispanic whites having the highest level of relevant health and hygiene knowledge, subsequently followed by Asian Americans, Hispanics, and non-Hispanic blacks [20]. Such differences may prevent ethnic minorities from taking appropriate countermeasures and may further exacerbate ethnic disparities in mortality and morbidity risk during the pandemic [20]. Besides, ethnic minorities are more likely to be faced with socioeconomic disadvantages, such as living in overcrowded places where it is difficult to keep physical distancing and holding essential jobs in social care and public transport where the risk of exposure is higher, which could also increase their mortality and morbidity [21, 22]. A recent study shows that African Americans have at least 50% higher odds than whites of working in essential industry sectors that are often exposed to infection, such as hospitals, healthcare and social assistance, and animal slaughtering and processing [23]. Inefficiencies and inadequacies in the health care system are an important reason why the poor are kept away from health care. Support for universal health care and primary health care remains inadequate [24], especially for immigrants dominated by ethnic minorities. More than 46.7 million immigrants now live in the US, but 11 million of them are undocumented, so they are not entitled to public health care and funding for public services [25]. In Texas, 32% of undocumented immigrants live below the poverty line and 64% do not have health insurance [2]. Such evidence may highlight the potentially disproportionate risks that ethnicities and minorities face over a long period of time prior to the outbreak of the pandemic.

## Method

### Setting

The advantage of using the US sample is that the US is a multi-ethnic country. According to the latest statistics on the ethnic profile of the US [26], the most prevalent racial or ethnic group is the White alone non-Hispanic population at 57.8%. The Hispanic population is the second-largest racial or ethnic group, comprising 18.7% of the total population. The non-Hispanic black population total 44.78 million people (13.5%) in 2020. Asian Americans are now the fastest-growing major racial or ethnic group in the US, accounting for 6.1% of the total population in the same year.

### Data description

Open data from multiple sources are combined and matched for regression analysis in this study. Specifically, *novel coronavirus disease data (confirmed cases)* come from the New York Times that continuously tracks the daily real-time cumulative counts of coronavirus cases in the US at the county level.

*Data of vaccination at the county-level* come from the Center for Disease Control and Prevention (CDC) in the US, which discloses the vaccination coverage not only in the total population, but also among those aged > 12, > 18 and > 65 years.

*Population composition data of ethnic minority and socioeconomic factors at the county level* are collected from the County Health Rankings & Roadmap Program (CHRRP, 2020), which is initiated and conducted in collaboration by Robert Wood Johnson Foundation and University of Wisconsin and aims to improve health equity and promote evidence-based policy and practical approaches [27, 28]. The CHRRP synthesizes the multi-source national health surveillance data with the time span ranging between 2016–2019 from various governmental agencies.

The availability of data from well-equipped statistical and survey institutions above provides an opportunity to examine the association between population distribution and health risks during the COVID-19 pandemic.

### Variables

Dependent variables are the *confirmed cases of COVID-19* (standardized by population, taken in natural log) at the county level, and the *vaccination coverage* in total population and among those aged > 12, > 18, and > 65 years.

Independent variables are population distribution by ethnicities, including *population percentage of non-Hispanic black* (Mean = 9.47, S.D. = 14.55), *population percentage of Asian American* (Mean = 1.59, S.D. = 2.84), and *population percentage of Hispanics* (Mean = 9.66, S.D. = 13.74).

Covariates include the following variables. *Primary care physician* is measured by the number of primary care physicians per 100,000 population (Mean = 52.78, S.D. = 34.52). *Life expectancy at the county level* is measured by the average life expectancy of a county (Mean = 76.83, S.D. = 7.43). *Housing problem* is measured by the percentage of households with at least one of following problems including overcrowding, high housing costs, lack of kitchen, or plumbing facilities (Mean = 13.95, S.D. = 4.34). *Regions* are classified into four categories according to two aspects (1) the median household income, and (2) the rich-poor polarization (measured by the income ratio of 80^th^ and 20^th^ percentile in a certain county). These four categories include Category-1, if the rich-poor polarization is above the average level and the median household income is above the average level; Category-2, if the rich-poor polarization is below the average level and the median household income is above the average level; Category-3, if the rich-poor polarization is above the average level and the median household income is below the average level; and Category-4, if the rich-poor polarization is below the average level and the median household income is below the average level.

### Statistical data analysis

Multiple linear regressions adjusted with robust standard errors are conducted to examine the relationships between variables in this study. To capture the dynamics over time, multiple linear regressions at equidistant time points (i.e., *May 28*^*th*^* 2020—Jan 28*^*th*^* 2021, with one-month interval between each timepoint*) are used to examine the associations between population distribution by ethnicities and the confirmed cases of COVID-19. Still, multiple linear regressions at equidistant time points (i.e., *Jan 25*^*th*^*—Aug 25*^*th*^*, 2021, with one-month interval between each timepoint*) are used to examine the associations between population distribution by ethnicities and vaccination coverage during this pandemic.

Given that vaccination began in January, 2021, multiple linear regressions of confirmed cases at equidistant time points are set to end on Jan 28^th^, 2021. By this way, this study seeks to isolate the impact of vaccination on confirmed cases when estimating the influence of population distribution on confirmed cases. Stata 16.0 (Stata Corp. LLC., College Station, TX, USA) is applied in the analysis. The regressions are shown as below.

$$Confirmed\;cases\;of\;COVID-19\;at\;the\;county\;level\;(standardized\;by\;population,\;taken\;in\;natural\;log)\;t=\beta0+\beta1\;Population\;percentage\;of\;non-Hispanic\;black\;+\;\beta2\;Population\;percentage\;of\;Asian\;American\;+\;\beta3\;Population\;percentage\;of\;Hispanics\;+\;\beta4\;Primary\;care\;physician\;per\;100,000\;population\;+\;\beta5\;Life\;expectancy\;at\;the\;county\;level\;+\beta6\;Percentage\;of\;households\;with\;housing\;problem\;+\;\beta7\;Category\;of\;regions\;+\;\varepsilon t$$

Where the subscript *t* indicates the *t-*th time point, including May 28^th^ 2020, Jun 28^th^, Jul 28^th^, Aug 28^th^, Sep 28^th^, Oct 28^th^, Nov 28^th^, Dec 28^th^ 2020, Jan. 28^th^ 2021.

$$Vaccination\;coverage\;at\;the\;county\;level\;(for\;different\;age\;groups\;including\;total\;population\;and\;>12,\;>18,\;and\;>65years)\;t=\;\beta0\;+\;\beta1\;Population\;percentage\;of\;non-Hispanicblack\;+\;\beta2\;Population\;percentage\;of\;Asian\;American+\;\beta3\;Population\;percentage\;of\;Hispanics\;+\;\beta4\;Primary\;care\;physician\;per\;100,000population\;+\;\beta5\;Life\;expectancy\;at\;the\;county\;level\;+\;\beta6\;Percentage\;of\;households\;with\;housing\;problem\;+\;\beta7\;Category\;of\;regions\;+\;\varepsilon t$$

Where the subscript *t* indicates the *t-*th time point, including Jan 25^th^ 2021, Feb 25^th^, Mar 25^th^, Apr 25^th^, May 25^th^, Jun 25^th^, Jul 25^th^, Aug 28^th^ 2021.

## Empirical results

**Fig. 1:**
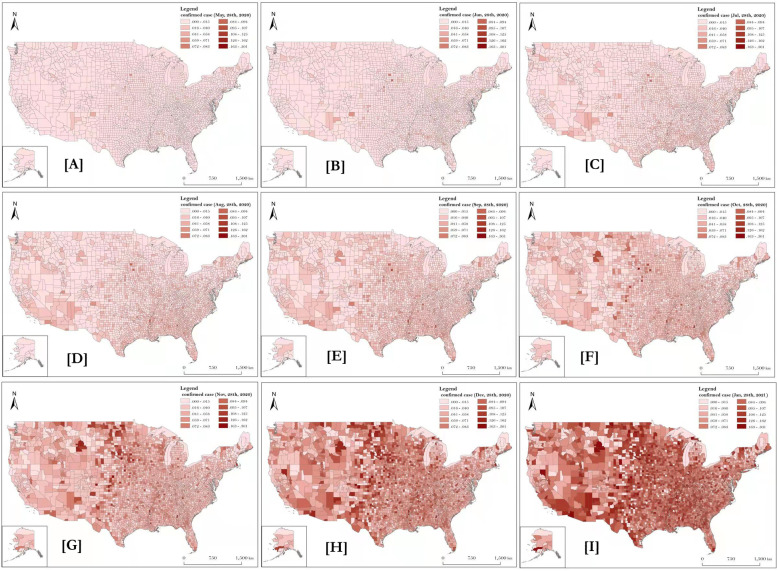
The distribution of COVID-19 cases on 28^th^ May 2020—28^th^ Jan 2021 (1A—1I)

The spatial distributions of confirmed cases (standardized by population, and the dynamics with time) are illustrated (see Fig. 1 in Appendix). Results of Table 1 demonstrate that the population percentage of ethnic minorities in a county (i.e., the non-Hispanic blacks and Hispanics) was positively associated with the confirmed cases (for the non-Hispanic blacks, 0.004–0.041, *p* < 0.01 from May 2020 to Jan 2021; for the Hispanics, 0.006–0.023, *p* < 0.01 from May 2020 to Jan 2021).

**Table 1:** The influences of population distribution by ethnic minority on confirmed cases of novel coronavirus disease in the US (county level)

	*Dependent variable: ln confirmed cases of COVID-19 (standardized by population)*																	
	*May. 28*^*th*^*, 2020*		*Jun. 28*^*th*^*, 2020*		*Jul. 28*^*th*^*, 2020*		*Aug. 28*^*th*^*, 2020*		*Sep. 28*^*th*^*, 2020*		*Oct. 28*^*th*^*, 2020*		*Nov. 28*^*th*^*, 2020*		*Dec. 28*^*th*^*, 2020*		*Jan. 28*^*th*^*, 2021*	
	*Coef*	*S.D*	*Coef*	*S.D*	*Coef*	*S.D*	*Coef*	*S.D*	*Coef*	*S.D*	*Coef*	*S.D*	*Coef*	*S.D*	*Coef*	*S.D*	*Coef*	*S.D*
**Independent variables**																		
*% Non-Hispanic black*	0.041^**^	0.002	0.041^**^	0.001	0.037^**^	0.001	0.032^**^	0.001	0.026^**^	0.001	0.018^**^	0.001	0.008^**^	0.001	0.004^**^	0.001	0.005^**^	0.001
*% Hispanics*	0.009^**^	0.002	0.018^**^	0.002	0.023^**^	0.001	0.020^**^	0.001	0.016^**^	0.001	0.011^**^	0.001	0.008^**^	0.001	0.007^**^	0.001	0.006^**^	0.001
*% Asian American*	0.042^**^	0.012	0.027^*^	0.011	0.010	0.008	0.009	0.006	0.006	0.006	-0.004	0.006	-0.018^**^	0.005	-0.018^**^	0.005	-0.018^**^	0.005
**Covariates**																		
*Regions*																		
*Category-1 high rich-poor polarization and high median household income*	REF		REF		REF		REF		REF		REF		REF		REF		REF	
*Category-2 low rich-poor polarization and high median household income*	0.107	0.064	0.106^*^	0.054	0.034	0.043	0.018	0.036	0.010	0.036	0.046	0.036	0.119^**^	0.034	0.075^**^	0.029	0.035	0.024
*Category-3 high rich-poor polarization and low median household income*	-0.477^**^	0.066	-0.324^**^	0.057	-0.123^**^	0.046	0.016	0.040	0.070	0.039	0.139^**^	0.037	0.142^**^	0.034	0.124^**^	0.028	0.096^**^	0.024
*Category-4 low rich-poor polarization and low median household income*	-0.372^**^	0.077	-0.260^**^	0.070	-0.211^**^	0.057	-0.118^*^	0.049	-0.096^*^	0.048	-0.005	0.046	0.081^*^	0.039	0.076^*^	0.032	0.038	0.028
*Primary care physicians per 100,000*	0.000	0.001	0.000^**^	0.001	-0.001	0.000	-0.002^**^	0.000	-0.002^**^	0.000	-0.001^**^	0.000	0.000	0.000	0.000	0.000	0.000	0.000
*Housing problems*	0.000	0.007	0.004^**^	0.006	0.002	0.005	-0.004	0.004	-0.013^**^	0.004	-0.022^**^	0.004	-0.028^**^	0.005	-0.027^**^	0.004	-0.022^**^	0.004
*Life expectancy at the county level*	-0.001	0.002	0.002^**^	0.002	0.003	0.002	0.002	0.002	0.003	0.002	0.003	0.002	0.001	0.002	0.000	0.002	0.000	0.002
*Intercept*	-6.837^**^	0.200	-6.560	0.182	-5.845^**^	0.180	-5.108^**^	0.149	-4.581^**^	0.158	-3.962^**^	0.180	-3.118^**^	0.135	-2.599^**^	0.125	-2.357^**^	0.127
*Num. of counties*	2950		2944		2944		2945		2945		2945		2945		2945		2945	
*F-statistics*	120.50		157.26		233.05		229.69		171.96		101.47		26.06		21.22		22.85	
*[p-value]*	[0.000]		[0.000]		[0.000]		[0.000]		[0.000]		[0.000]		[0.000]		[0.000]		[0.000]	

*Notes*: Given that the US has an even ratio of males to females, which is also shown on county level, there is no enough variation in gender ratio on county level to account for the variation in the dependent variable. For this reason, the gender ratio on county level was not included in the regression analysis. ^*^p < 0.05, ^**^p < 0.01

For the population percentage of Asian Americans, its association with confirmed cases was significantly positive in the early phase (0.042 in May 2020, and 0.027 in Jun 2020, *p* < 0.01), and insignificant in the mid-phase (0.010 in Jul 2020, 0.009 in Aug 2020, 0.006 in Sep 2020, -0.004 in Oct 2020, *p* > 0.10), and then significantly negative in the later phase (-0.018 in Nov 2020, -0.018 in Dec 2020, -0.018 in Jan 2021, *p* < 0.01). These results provide evidence for ethnic differences in the infection risk exposure during the pandemic.

**Fig. 2:**
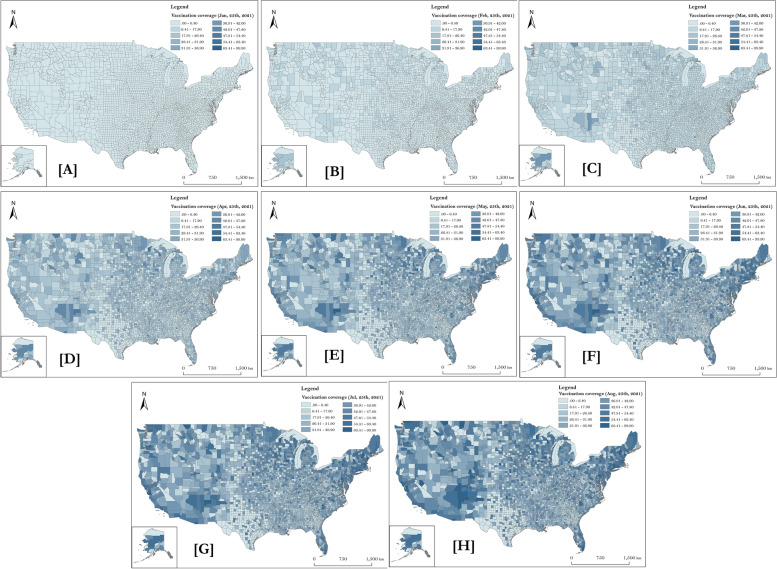
The distribution of vaccination coverage (for total population) on 25^th^ Jan 2021—25^th^ Aug 2021(2A—2H)

**Fig. 3:**
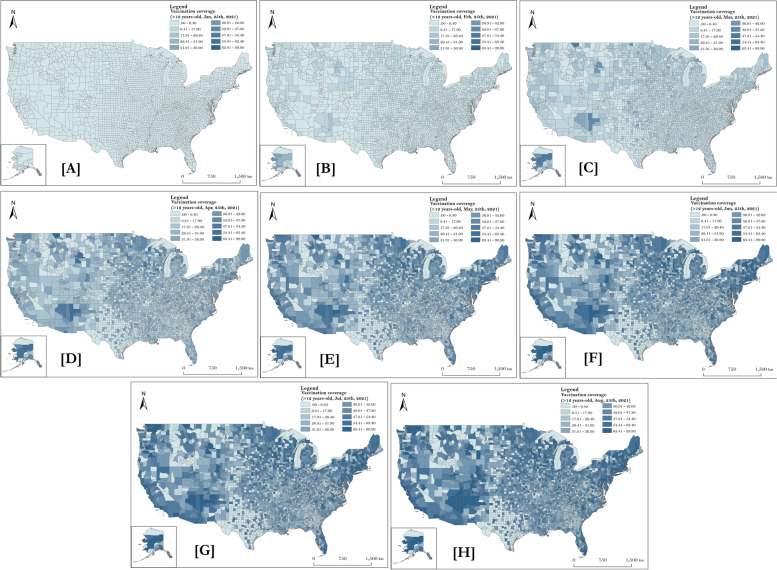
The distribution of vaccination coverage (for > 12 years old) on 25^th^ Jan 2021—25^th^ Aug 2021(3A—3H)

**Fig. 4:**
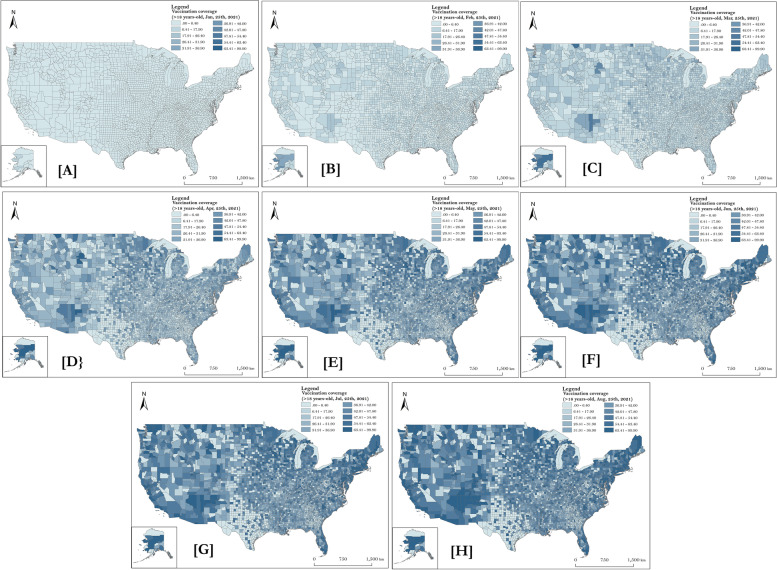
The distribution of vaccination coverage (for > 18 years old) on 25^th^ Jan 2021—25^th^ Aug 2021(4A—4H)

**Fig. 5:**
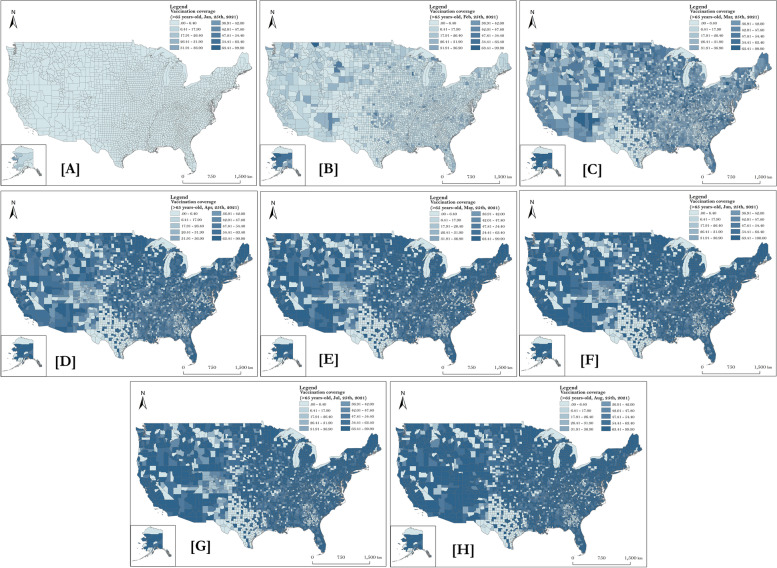
The distribution of vaccination coverage (for > 65 years old) on 25^th^ Jan 2021—25^th^ Aug 2021(5A—5H)

Besides, the spatial distributions of vaccination coverage (population percentage, and the dynamics with time, in total population and among those aged > 12, > 18, and > 65 years) are illustrated (see Figs. 2, 3, 4 and 5 in Appendix). As depicted in these figures, the population aged > 65 years outperform other age groups in vaccination coverage. Tables 2, 3, 4 and 5 show the associations between population distribution by ethnicities and vaccination coverage in total population and among those aged > 12, > 18, and > 65 years respectively. As shown in Table 2, there are significant negative associations between population distribution by non-Hispanic Black/Hispanics and vaccination coverage at all timepoints in the total population, which indicates that counties with a larger proportion of non-Hispanic Black/Hispanics in the total population have a lower level of vaccination coverage. Similar support is found for the population distribution by non-Hispanic Black/Hispanics across different age groups. In Tables 3, 4 and 5, counties with a larger proportion of non-Hispanic Black/Hispanics in age groups > 12, > 18, and > 65 years also show lower vaccination coverage at most timepoints. Further, as shown in Table 2, 3, 4 and 5, the associations between population distribution by Asian Americans and vaccination coverage at most timepoints and in most age groups are insignificant. These results indicate that there are no associations between population distribution by Asian Americans and vaccination coverage.

**Table 2:** The influences of population distribution by ethnic minority on COVID-19 vaccination coverage (*total population*) in the US (county level)

	*Dependent variable: Vaccination coverage*															
	*Jan. 25*^*th*^*, 2021*		*Feb. 25*^*th*^*, 2021*		*Mar. 25*^*th*^*, 2021*		*Apr. 25*^*th*^*, 2021*		*May. 25*^*th*^*, 2021*		*Jun. 25*^*th*^*, 2021*		*Jul. 25*^*th*^*, 2021*		*Aug. 25*^*th*^*, 2021*	
	*Coef*	*S.D*	*Coef*	*S.D*	*Coef*	*S.D*	*Coef*	*S.D*	*Coef*	*S.D*	*Coef*	*S.D*	*Coef*	*S.D*	*Coef*	*S.D*
**Independent variables**																
*% Non-Hispanic black*	-0.009^**^	0.001	-0.034^**^	0.005	-0.081^**^	0.009	-0.126^**^	0.014	-0.146^**^	0.018	-0.155^**^	0.019	-0.151^**^	0.020	-0.145^**^	0.022
*% Hispanics*	-0.012^**^	0.001	-0.097^**^	0.005	-0.179^**^	0.009	-0.298^**^	0.016	-0.359^**^	0.021	-0.388^**^	0.024	-0.399^**^	0.026	-0.400^**^	0.028
*% Asian American*	-0.004	0.008	-0.050	0.030	-0.111	0.058	-0.060	0.113	0.174	0.173	0.272	0.205	0.306	0.218	0.267	0.226
**Covariates**																
*Regions*																
*Category-1 high rich-poor polarization and high median household income*	REF		REF		REF		REF		REF		REF		REF		REF	
*Category-2 low rich-poor polarization and high median household income*	0.064	0.051	0.010	0.200	0.214	0.355	0.569	0.597	1.081	0.770	1.436	0.876	1.590	0.921	1.936^*^	0.965
*Category-3 high rich-poor polarization and low median household income*	-0.072	0.046	-0.108	0.191	-0.840^*^	0.359	-2.831^**^	0.621	-5.024^**^	0.805	-5.988^**^	0.913	-6.270^**^	0.962	-6.277^**^	1.009
*Category-4 low rich-poor polarization and low median household income*	-0.067	0.048	0.071	0.238	0.053	0.423	-1.226	0.705	-2.532^**^	0.879	-3.099^**^	0.983	-3.229^**^	1.030	-3.236^**^	1.070
*Primary care physicians per 100,000*	0.006^**^	0.001	0.017^**^	0.003	0.034^**^	0.005	0.063^**^	0.007	0.087^**^	0.010	0.099^**^	0.011	0.102^**^	0.012	0.108^**^	0.013
*Housing problems*	0.034^**^	0.013	0.138^**^	0.037	0.162^**^	0.049	0.279^**^	0.053	0.454^**^	0.064	0.600^**^	0.074	0.658^**^	0.079	0.733^**^	0.085
*Life expectancy at the county level*	0.001	0.002	0.024^**^	0.009	0.059^**^	0.017	0.134^**^	0.026	0.183^**^	0.032	0.201^**^	0.036	0.204^**^	0.039	0.182^**^	0.053
*Intercept*	-0.076	0.152	1.777^*^	0.750	6.290^**^	1.394	9.622^**^	2.146	9.194^**^	2.656	8.937^**^	2.974	9.464^**^	3.154	11.762^**^	4.098
*Num. of counties*	2950		2950		2950		2950		2950		2950		2950		2950	
*F-statistics*	49.72		88.49		82.30		88.51		101.46		100.96		96.53		87.20	
*[p-value]*	[0.000]		[0.000]		[0.000]		[0.000]		[0.000]		[0.000]		[0.000]		[0.000]	

*Notes*: Given that the US has an even ratio of males to females, which is also shown on county level, there is no enough variation in gender ratio on county level to account for the variation in the dependent variable. For this reason, the gender ratio on county level was not included in the regression analysis. This consideration is also applied to the regression analysis in Table 3–5. ^*^p < 0.05, ^**^p < 0.01

**Table 3:** The influences of population distribution by ethnic minority on COVID-19 vaccination coverage (> *12 years of age*) in the US (county level)

	*Dependent variable: Vaccination coverage in population* > *12 years of age*															
	*Jan. 25*^*th*^*, 2021*		*Feb. 25*^*th*^*, 2021*		*Mar. 25*^*th*^*, 2021*		*Apr. 25*^*th*^*, 2021*		*May. 25*^*th*^*, 2021*		*Jun. 25*^*th*^*, 2021*		*Jul. 25*^*th*^*, 2021*		*Aug. 25*^*th*^*, 2021*	
	*Coef*	*S.D*	*Coef*	*S.D*	*Coef*	*S.D*	*Coef*	*S.D*	*Coef*	*S.D*	*Coef*	*S.D*	*Coef*	*S.D*	*Coef*	*S.D*
**Independent variables**																
*% Non-Hispanic black*	-0.011^**^	0.002	-0.042^**^	0.006	-0.095^**^	0.011	-0.147^**^	0.017	-0.168^**^	0.021	-0.178^**^	0.023	-0.173^**^	0.024	-0.165^**^	0.025
*% Hispanics*	-0.015^**^	0.002	-0.113^**^	0.006	-0.208^**^	0.011	-0.343^**^	0.019	-0.411^**^	0.025	-0.443^**^	0.029	-0.455^**^	0.031	-0.455^**^	0.033
*% Asian American*	-0.008	0.010	-0.065	0.037	-0.130	0.070	-0.069	0.132	0.207	0.203	0.318	0.240	0.357	0.255	0.312	0.264
**Covariates**																
*Regions*																
*Category-1 high rich-poor polarization and high median household income*	REF		REF		REF		REF		REF		REF		REF		REF	
*Category-2 low rich-poor polarization and high median household income*	0.088	0.063	0.116	0.243	0.456	0.423	1.035	0.701	1.732	0.899	2.196^*^	1.021	2.400^*^	1.075	2.818^*^	1.125
*Category-3 high rich-poor polarization and low median household income*	-0.092	0.056	-0.074	0.228	-0.859^*^	0.424	-3.085^**^	0.727	-5.609^**^	0.940	-6.730^**^	1.066	-7.063^**^	1.124	-7.084^**^	1.179
*Category-4 low rich-poor polarization and low median household income*	-0.072	0.058	0.161	0.286	0.120	0.504	-1.360	0.814	-2.852^**^	1.019	-3.495^**^	1.141	-3.642^**^	1.197	-3.656^**^	1.247
*Primary care physicians per 100,000*	0.007^**^	0.001	0.020^**^	0.003	0.039^**^	0.005	0.072^**^	0.009	0.099^**^	0.011	0.112^**^	0.013	0.116^**^	0.013	0.123^**^	0.014
*Housing problems*	0.046^**^	0.017	0.181^**^	0.051	0.210^**^	0.065	0.346^**^	0.067	0.548^**^	0.075	0.718^**^	0.085	0.786^**^	0.089	0.875^**^	0.095
*Life expectancy at the county level*	0.001	0.002	0.028^**^	0.010	0.066^**^	0.020	0.156^**^	0.030	0.211^**^	0.037	0.231^**^	0.042	0.234^**^	0.045	0.208^**^	0.060
*Intercept*	-0.119	0.193	1.768	0.907	7.156^**^	1.682	10.754^**^	2.475	10.370^**^	3.082	10.157^**^	3.458	10.803^**^	3.677	13.433^**^	4.707
*Num. of counties*	2916		2916		2916		2916		2916		2916		2916		2916	
*F-statistics*	47.18		83.78		77.69		85.37		100.38		101.09		272.64		88.02	
*[p-value]*	[0.000]		[0.000]		[0.000]		[0.000]		[0.000]		[0.000]		[0.000]		[0.000]	

*Notes*: The regression analysis is based on the sample where all of variables have no missing values. Therefore, the valid sample size (the number of counties) in Table 3 has some discrepancies with values reported in Table 4 & 5. ^*^p < 0.05, ^**^p < 0.01

**Table 4:** The influences of population distribution by ethnic minority on COVID-19 vaccination coverage (> *18 years of age*) in the US (county level)

	*Dependent variable: Vaccination coverage in population* > *18 years of age*															
	*Jan. 25*^*th*^*, 2021*		*Feb. 25*^*th*^*, 2021*		*Mar. 25*^*th*^*, 2021*		*Apr. 25*^*th*^*, 2021*		*May. 25*^*th*^*, 2021*		*Jun. 25*^*th*^*, 2021*		*Jul. 25*^*th*^*, 2021*		*Aug. 25*^*th*^*, 2021*	
	*Coef*	*S.D*	*Coef*	*S.D*	*Coef*	*S.D*	*Coef*	*S.D*	*Coef*	*S.D*	*Coef*	*S.D*	*Coef*	*S.D*	*Coef*	*S.D*
**Independent variables**																
*% Non-Hispanic black*	-0.012^**^	0.002	-0.046^**^	0.007	-0.105^**^	0.012	-0.160^**^	0.018	-0.180^**^	0.022	-0.183^**^	0.024	-0.175^**^	0.025	-0.171^**^	0.026
*% Hispanics*	-0.016^**^	0.002	-0.123^**^	0.007	-0.224^**^	0.012	-0.367^**^	0.021	-0.438^**^	0.027	-0.466^**^	0.030	-0.477^**^	0.032	-0.475^**^	0.034
*% Asian American*	-0.008	0.011	-0.073	0.041	-0.149^*^	0.076	-0.081	0.143	0.201	0.216	0.283	0.245	0.313	0.258	0.264	0.266
**Covariates**																
*Regions*																
*Category-1 high rich-poor polarization and high median household income*	REF		REF		REF		REF		REF		REF		REF		REF	
*Category-2 low rich-poor polarization and high median household income*	0.108	0.070	0.158	0.266	0.580	0.460	1.252	0.760	2.047^*^	0.963	2.482^*^	1.061	2.637^*^	1.109	3.044^**^	1.153
*Category-3 high rich-poor polarization and low median household income*	-0.096	0.062	-0.110	0.250	-0.984^*^	0.462	-3.463^**^	0.790	-6.059^**^	1.008	-6.922^**^	1.111	-7.205^**^	1.162	-7.175^**^	1.210
*Category-4 low rich-poor polarization and low median household income*	-0.084	0.064	0.083	0.307	0.042	0.542	-1.671	0.871	-3.194^**^	1.083	-3.584^**^	1.184	-3.688^**^	1.234	-3.652^**^	1.277
*Primary care physicians per 100,000*	0.008^**^	0.001	0.021^**^	0.003	0.041^**^	0.006	0.076^**^	0.009	0.103^**^	0.012	0.112^**^	0.013	0.114^**^	0.014	0.121^**^	0.015
*Housing problems*	0.051^*^	0.020	0.200^**^	0.057	0.229^**^	0.072	0.362^**^	0.074	0.568^**^	0.080	0.709^**^	0.087	0.766^**^	0.090	0.850^**^	0.095
*Life expectancy at the county level*	0.001	0.002	0.026^*^	0.012	0.067^**^	0.021	0.159^**^	0.032	0.218^**^	0.039	0.236^**^	0.042	0.239^**^	0.045	0.212^**^	0.059
*Intercept*	-0.133	0.216	2.344^*^	1.025	8.269^**^	1.786	12.915^**^	2.660	12.595^**^	3.213	12.659^**^	3.496	13.324^**^	3.687	15.929^**^	4.627
*Num. of counties*	2950		2950		2950		2950		2950		2950		2950		2950	
*F-statistics*	46.51		81.99		77.30		84.84		100.23		99.76		95.16		87.36	
*[p-value]*	[0.000]		[0.000]		[0.000]		[0.000]		[0.000]		[0.000]		[0.000]		[0.000]	

*Notes*: ^*^p < 0.05, ^**^p < 0.01

**Table 5:** The influences of population distribution by ethnic minority on COVID-19 vaccination coverage (> *65 years of age*) in the US (county level)

	*Dependent variable: Vaccination coverage in population* > *65 years of age*															
	*Jan. 25*^*th*^*, 2021*		*Feb. 25*^*th*^*, 2021*		*Mar. 25*^*th*^*, 2021*		*Apr. 25*^*th*^*, 2021*		*May. 25*^*th*^*, 2021*		*Jun. 25*^*th*^*, 2021*		*Jul. 25*^*th*^*, 2021*		*Aug. 25*^*th*^*, 2021*	
	*Coef*	*S.D*	*Coef*	*S.D*	*Coef*	*S.D*	*Coef*	*S.D*	*Coef*	*S.D*	*Coef*	*S.D*	*Coef*	*S.D*	*Coef*	*S.D*
**Independent variables**																
*% Non-Hispanic black*	-0.014^**^	0.004	-0.009	0.016	-0.108^**^	0.024	-0.200^**^	0.031	-0.201^**^	0.033	-0.200^**^	0.034	-0.193^**^	0.035	-0.212^**^	0.036
*% Hispanics*	-0.017^**^	0.005	-0.227^**^	0.014	-0.490^**^	0.025	-0.712^**^	0.035	-0.765^**^	0.038	-0.788^**^	0.040	-0.798^**^	0.041	-0.770^**^	0.042
*% Asian American*	-0.032	0.022	-0.006	0.095	0.060	0.191	-0.002	0.259	-0.001	0.282	0.014	0.294	0.014	0.301	-0.125	0.301
**Covariates**																
*Regions*																
*Category-1 high rich-poor polarization and high median household income*	REF		REF		REF		REF		REF		REF		REF		REF	
*Category-2 low rich-poor polarization and high median household income*	0.198	0.152	-0.552	0.631	0.719	1.053	2.472	1.346	3.025^*^	1.427	3.225^*^	1.474	3.327^*^	1.503	4.214^**^	1.502
*Category-3 high rich-poor polarization and low median household income*	-0.039	0.089	0.047	0.649	-4.025^**^	1.091	-6.657^**^	1.401	-7.478^**^	1.499	-7.679^**^	1.548	-7.847^**^	1.578	-7.594^**^	1.596
*Category-4 low rich-poor polarization and low median household income*	0.037	0.104	-0.360	0.726	-2.267	1.186	-3.779^*^	1.505	-3.869^*^	1.602	-3.863^*^	1.653	-3.897^*^	1.684	-3.696^*^	1.696
*Primary care physicians per 100,000*	0.004^*^	0.002	0.017^*^	0.007	0.060^**^	0.012	0.087^**^	0.016	0.095^**^	0.017	0.097^**^	0.017	0.097^**^	0.018	0.112^**^	0.018
*Housing problems*	0.101^*^	0.047	0.357	0.095	0.288^**^	0.102	0.520^**^	0.109	0.647^**^	0.118	0.704^**^	0.123	0.738^**^	0.125	0.875^**^	0.126
*Life expectancy at the county level*	-0.004	0.003	0.039	0.026	0.197^**^	0.042	0.318^**^	0.056	0.340^**^	0.061	0.347^**^	0.063	0.346^**^	0.065	0.309^**^	0.070
*Intercept*	-0.485	0.427	6.924	2.161	20.511^**^	3.494	26.665^**^	4.639	27.781^**^	5.008	28.590^**^	5.183	29.530^**^	5.361	31.294^**^	5.597
*Num. of counties*	2950		2950		2950		2950		2950		2950		2950		2950	
*F-statistics*	11.13		48.05		71.33		87.83		88.82		86.81		84.05		82.64	
*[p-value]*	[0.000]		[0.000]		[0.000]		[0.000]		[0.000]		[0.000]		[0.000]		[0.000]	

*Notes*: ^*^p < 0.05, ^**^p < 0.01

## Discussion

This study provided an empirical examination on the relationships between population distribution by ethnicity and infection risk/vaccination coverage during the pandemic from the population composition perspective. In practice, it could help policymakers formulate effective plans for the geographical distribution of healthcare resources to improve the health care access of vulnerable groups in health crises.

Results of this study provided macro-level evidence for previous studies that reported the inequity in health risks for ethnic minorities during this pandemic. This study revealed that the population distributions of non-Hispanic blacks and Hispanics are shown stably and positively associated with the confirmed cases in all cross-sections from May, 2020 to Jan, 2021, which could confirm previous findings reporting the disproportionate infection risk to ethnic minorities such as the non-Hispanic Black during the pandemic [14–17]. In addition, results of this study suggested that the negative association between population distribution of ethnic minorities and health disparities can extend from infection risk to health care utilization/access during the pandemic. Areas with higher population distribution of non-Hispanic Black/Hispanics displayed a lower level of vaccination coverage in this study. This finding worked not just for total population, but also for subgroups aged > 12, > 18 and > 65 years. Some potential causes may lead to a higher risk of infection and less access to vaccination for ethnic minorities. To be specific, this groups of people is often faced with poorer socioeconomic status [21, 22, 29, 30]. They have more chance of having essential jobs with higher exposure risks [5, 23, 31–33], but have less access to health services [4, 6, 12]. They are also more likely to suffer from inequity in pre-existing diseases and stigmatization [18, 19, 34], and to lack adequate health awareness, information, and knowledge about the novel coronavirus disease pandemic [9, 20, 35].

The population aged > 65 years are shown to outperform other age groups in vaccination coverage (see Figures). This higher vaccination coverage is consistent with the prioritization of this age group early in the vaccination program. Following recommendations by the Advisory Committee on Immunization Practices to prioritize COVID-19 vaccination for specific groups of the US population at highest risk for COVID-19 hospitalization and death, many states included older adults among the first groups eligible for vaccination [36]. However, vaccination coverage for those aged > 65 years still varies by race and ethnicity. Among recipients aged > 65 years of at least 1 dose of COVID-19 vaccine, 70.7% were White, 7.6% were non-Hispanic Black, 6.7% were Hispanic, and 3.5% were Asian American [37].

In contrast, the association between the population distribution of Asian Americans and the confirmed cases showed a transition from positive to negative in this study. The association between population distribution of Asian Americans and vaccination coverage did not appear significant as well. Both results may indicate that Asian Americans are better adapted to the requirements of health care practice in the pandemic, though a higher level of confirmed cases among Asian Americans during the early stage of pandemic outbreak because of social vulnerability in racial/ethnic minority groups. These results confirmed prior findings that Asian Americans performed better on COVID-19 knowledge, attitudes and health literacy in the US [38], and had lower hospitalization rates than other minorities in a survey of 12 states [39].

This study has some policy implications. First, the characteristics of population distribution should be considered in epidemic prevention and control, which is of great importance for public health management in a region or even a country. The regions whose population distribution reflects a high risk of infection (and low vaccination acceptance) should be focused. Additional assistance, such as the increase in welfare and food supplies and the reduction in racial segregation, is particularly important for these regions during the pandemic. Second, the improvement of medical and testing conditions is the key practices to reduce inequity in healthcare resources in these regions. Government agencies should increase the necessary investments in medical resources, expand the coverage of health insurance, establish more medical aid centers, equip more professional medical personnel, and provide timely and free nucleic acid testing, etc. besides non-pharmaceutical intervention (e.g., lockdown [40, 41], social distancing). Third, the disparities in vulnerability to this pandemic between regions with different population distribution structures highlight deep-rooted social problems that need to be addressed for the long-term development of society. Forth, continued efforts are needed to improve vaccination coverage among persons in the group aged under 65 years. Community-specific messaging could engage this age group by using trusted sources to explain the value of vaccination to communities and individuals and to address concerns about vaccine safety. In addition, this age group might be reached by setting up strategically-located mobile and walk-in clinics with flexible hours, providing vaccinations in the workplace, and encouraging employers to provide paid leave for employees to receive the vaccine and treat any vaccine-related side effects.

There are still some limitations in this study. First, this study treated the population distribution as static, since it is stable in a short period of time. Due to unavailability of data, this study could not include the dynamic characteristics of population distribution on a county level in daily/monthly frequency to investigate their associations with the pandemic development. With relevant data available, future research need to provide a comprehensive investigation. Moreover, in the absence of data on the population distribution and the pandemic development at a lower regional level, this study focused mainly on the county-level relationships. With data at a lower regional level available, future research can conduct a fine-grained investigation, such as at the community level, to make research findings of more practical significance. Furthermore, the availability of data limited the investigation in other multi-ethnic countries outside the US. Future research can make comparative analysis when other data sources are available.

## Conclusions

The population distribution by racial/ethnic minority is shown, on county level, associated with the infection risk and vaccination coverage in the US during the COVID-19 pandemic. The findings provide a macro-level identifier of health risks for epidemic prevention and control, and provide scientific support for reducing inequity in health risks of vulnerable populations and promoting regional public health management. It is therefore essential to pay close attention to the regions whose population distribution reflects a higher health risk and take measures to reduce inequity faced by vulnerable populations in terms of health resources, social security, racial and social issues. Further research needs to make a more fine-grained exploration of the relationships between population distribution and regional health risks at lower regional levels and in the contexts of other multi-ethnic countries with richer characteristics of population distribution, in order to provide more operational and universal guidance.

## Data Availability

The novel coronavirus disease data come from the New York Times novel coronavirus disease tracking project (https://github.com/nytimes/covid-19-data). The data of social-economic factors related to children and youth on county level are collected from the County Health Rankings & Roadmap Program (2020). The data of vaccination coverage are publicly accessible from CDC.

## Appendix

Figure 1 depicts the spatial distribution and dynamic changes of confirmed cases over time. Figs. 2, 3, 4 and 5 depict the spatial distribution and dynamics of vaccine coverage over time for the total population and those aged > 12, > 18, and > 65 years respectively. As depicted in Fig. 1, the legends in different shades of colors on the upper right show the different levels of confirmed cases standardized by population. The larger the legend value (or the darker the color), the greater the level of confirmed cases in a specific place at a given timepoint. Similarly, as depicted in Fig. 2, the legends in different shades of colors on the upper right show the different levels of vaccine coverage. The larger the legend value (or the darker the color), the greater the level of vaccine coverage in total population in a specific place at a given timepoint. The same goes for the description of legends in Figs. 3, 4 and 5.

## Abbreviations

CHRRP: County Health Rankings & Roadmap Program
CDC: Center for Disease Control and Prevention
COVID-19: Coronavirus disease 2019
HIV: Human immunodeficiency virus
